# Expanding the genetic code of *Mus musculus*

**DOI:** 10.1038/ncomms14568

**Published:** 2017-02-21

**Authors:** Songmi Han, Aerin Yang, Soonjang Lee, Han-Woong Lee, Chan Bae Park, Hee-Sung Park

**Affiliations:** 1 Department of Physiology, Ajou University School of Medicine, Suwon 16499, Republic of Korea; 2 Department of Chemistry, Korea Advanced Institute of Science and Technology, 291 Daehak-ro, Yuseong-gu, Daejeon 34141, Republic of Korea; 3 Department of Biochemistry, Yonsei Laboratory Animal Research Center, Yonsei University, Seoul 03722, Republic of Korea

## Abstract

Here we report the expansion of the genetic code of *Mus musculus* with various unnatural amino acids including *N*^ɛ^-acetyl-lysine. Stable integration of transgenes encoding an engineered *N*^ɛ^-acetyl-lysyl-tRNA synthetase (AcKRS)/tRNA^Pyl^ pair into the mouse genome enables site-specific incorporation of unnatural amino acids into a target protein in response to the amber codon. We demonstrate temporal and spatial control of protein acetylation in various organs of the transgenic mouse using a recombinant green fluorescent protein (GFPuv) as a model protein. This strategy will provide a powerful tool for systematic *in vivo* study of cellular proteins in the most commonly used mammalian model organism for human physiology and disease.

## Abstract (web summary)

Expanding the genetic code with unnatural amino acids in model organisms is a powerful tool for investigating *in vivo* cellular proteins and processes. Here the authors re-engineer the amber stop codon in laboratory mice to allow site-specific incorporation of unnatural amino acids in target proteins.

Post-translational modifications are crucial for regulating protein function, and thus play a key role in many essential cellular processes^1^. The amber codon suppression technique, based on the use of an orthogonal aminoacyl-tRNA synthetase/tRNA pair, has been successfully developed as a means to expand a protein's functionalities in the laboratory^2^. This approach has been widely used to examine various aspects of proteins at the molecular and cellular level^3^. Thus far, this technique has been efficiently applied to expand the genetic code of diverse organisms ranging from bacteria^4^, yeast^5^, mammalian^6^, stem cells^7^ and neurons^8^ to multicellular organisms including a primitive animal^9^, insect^10^ and plant^11^. However, despite extensive efforts, this powerful approach has not been extended to the multiorgan animal *Mus musculus*, the most prevalent model of human physiology and disease. The mouse is the model organism whose genome is most closely related to that of humans: its genome is more than 99% similar to that of humans, and it contains most human gene counterparts or functionally related genes^12^. The mouse has short life span and is easy to breed and handle in the laboratory. More importantly, the mouse genome is readily manipulated, which makes it possible to generate custom-made mutant mouse strains, enabling detailed *in vivo* study of specific genes and providing excellent models for various human diseases^13^. Thus, the mouse has become the premier mammalian model system for basic and applied biomedical research. Here we report for the first time the generation of a mouse strain with an expanded genetic code, allowing site-specific incorporation of unnatural amino acids (UAAs) including *N*^ɛ^-acetyl-lysine (AcK) in response to the amber codon in various organs. We also demonstrate that our approach enables rapid onset of position-specific acetylation of a target protein at any developmental stage or selected tissue, facilitating temporal and spatial control of protein acetylation in the transgenic mouse.

## Results

### Construction of AcK-incorporation system

**Figure 1:**
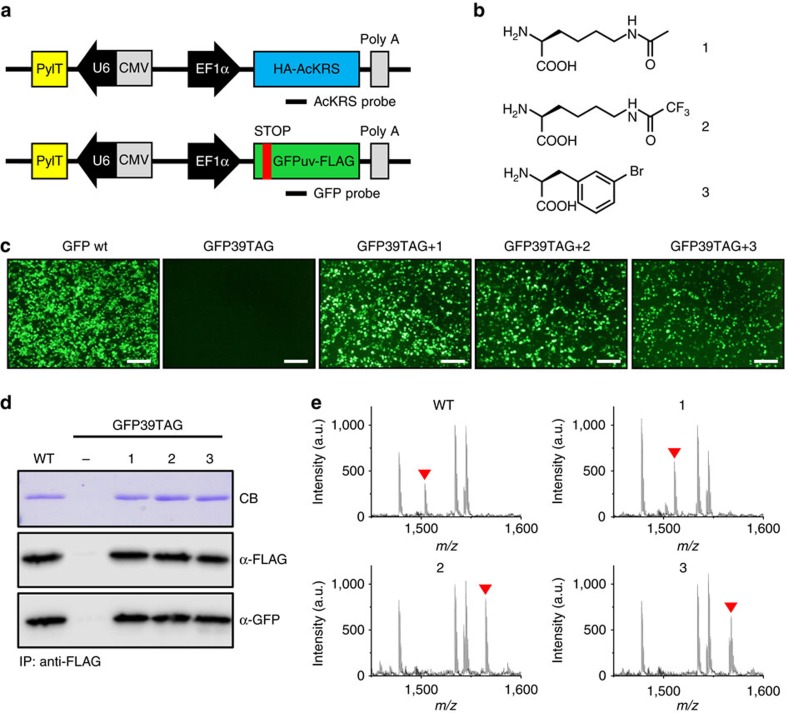
Construction and validation of the AcK-incorporation system. (**a**) Schematic diagrams of the DNA constructs, *PylT::HA-AcKRS* and *PylT::GFP39TAG-FLAG*. Probes used for Southern blotting of the transgenic mouse are indicated. (**b**) UAAs used in this study: *N*^ɛ^-acetyl-lysine (1, AcK), *N*^ɛ^-trifluoroacetyl-lysine (2, tfAcK) and 3-bromo-phenylalanine (3, BrF). (**c**) Fluorescence image of HEK293T cells transfected with DNA constructs and cultured in the presence of UAA, AcK, tfAcK or BrF. Scale bar, 200 μm. (**d**) Western blot analysis of anti-FLAG–immunoprecipitated proteins from the lysates of the transfected cells, using antibodies against FLAG-tag and GFPuv. (**e**) MALDI-TOF MS analysis of proteins after trypsin digestion. MS analysis of trypsin-treated GFPuv samples clearly illustrates the incorporation of the UAA (AcK, tfAcK or BrF) into the designated position of the model protein. The peak corresponding to the tryptic peptide carrying Tyr or the UAA at position 39 of GFPuv is indicated (arrowhead). The mass of Y39-containing tryptic peptide (GFP wt) is 1503.7 Da (calculated mass; 1,503.7 Da). The detected mass of AcK39-containing peptide (GFP-39AcK) is 1,510.9 Da (expected mass; 1,510.7 Da), that of tfAcK39-carrying peptide is 1,564.6 Da (expected mass; 1,564.7 Da) and that of BrF-carrying peptide (GFP-39BrF) is 1,565.8 Da (expected mass; 1,565.6 Da).

Lysine acetylation is a reversible post-translational modification that dynamically regulates functions of a wide range of eukaryotic proteins; thus, it critically affects numerous cellular processes^14^. In particular, aberrant acetylation of many cellular proteins is associated with various human diseases, including cancer^15^. However, detailed functional analyses of protein acetylation have been hampered by technical difficulties in controlling acetylation in an animal system. Recently, we and others have developed efficient methods enabling selective chemical modifications in proteins^16^,^17^, including phosphorylation^18^ and acetylation^19^. To establish an animal model that allows temporal and spatial control of acetylation, we sought to construct a transgenic mouse with an expanded genetic code that enables site-specific incorporation of AcK in response to the amber codon. To this end, we selected an engineered *N*^ɛ^-acetyl-lysyl-tRNA synthetase (AcKRS)/tRNA^Pyl^ pair previously developed in bacteria^20^. AcKRS is also known to aminoacylate various UAAs^21^,^22^. For the AcK-incorporation system, we chose to use ubiquitous promoters. The impact of protein acetylation on cellular function differs depending on the tissue type as well as developmental stage, an issue that can only be addressed at the whole-animal level. Accordingly, we constructed a pAcKRS-tRNA plasmid in which the expression of HA-tagged AcKRS is controlled by the constitutive human elongation factor 1α (EF1α) promoter (Fig. 1a and Supplementary Fig. 1). The transcription of tRNA^Pyl^ is driven by the RNA polymerase III promoter U6 and the human cytomegalovirus (CMV) immediate early region enhancer (Fig. 1a). To demonstrate the utility of the AcK-incorporation system, we used recombinant green fluorescent protein (GFPuv) as a model protein, again using the constitutive EF1α promoter to drive expression of FLAG-tagged GFPuv carrying an amber stop codon at position 39. Another copy of the tRNA^Pyl^ expression cassette was inserted into the GFPuv-expressing plasmid pGFPamber-tRNA (Fig. 1a and Supplementary Fig. 2).

### Validation of AcK incorporation in mammalian cells

To demonstrate the feasibility of the generated constructs, we first co-transfected the plasmids pAcKRS-tRNA and pGFPamber-tRNA into the mammalian cell line, HEK293T, and validated AcK incorporation in response to the amber codon in GFPuv. GFPuv fluorescence signals were detected only in cells incubated with AcK, and not in those without it (Fig. 1b,c). We also tested two other UAAs, *N*^ɛ^-trifluoroacetyl-lysine (tfAcK) and 3-bromo-phenylalanine (BrF; Fig. 1b). Clear GFPuv fluorescence was detected only in the presence of AcK, tfAcK or BrF (Fig. 1c). Western blotting analyses using anti-FLAG and anti-GFP antibodies revealed that the expression of GFPuv is clearly dependent upon the presence of these UAAs (Fig. 1d and Supplementary Fig. 3). Finally, MALDI-TOF MS analysis after in-gel trypsin digestion of the proteins clearly confirmed site-specific incorporation of these UAAs in response to the amber codon (Fig. 1e, Supplementary Fig. 4, and Supplementary Tables 1 & 2). Similarly, NIH3T3 mouse cells transfected with an AcK-incorporation system expressed GFPuv only in the presence of AcK (Supplementary Fig. 5a). western blotting and MALDI-TOF MS analysis demonstrated the site-specific incorporation of AcK into GFPuv in this mouse cell line (Supplementary Fig. 5b,c).

### Generation of a transgenic mouse

**Figure 2:**
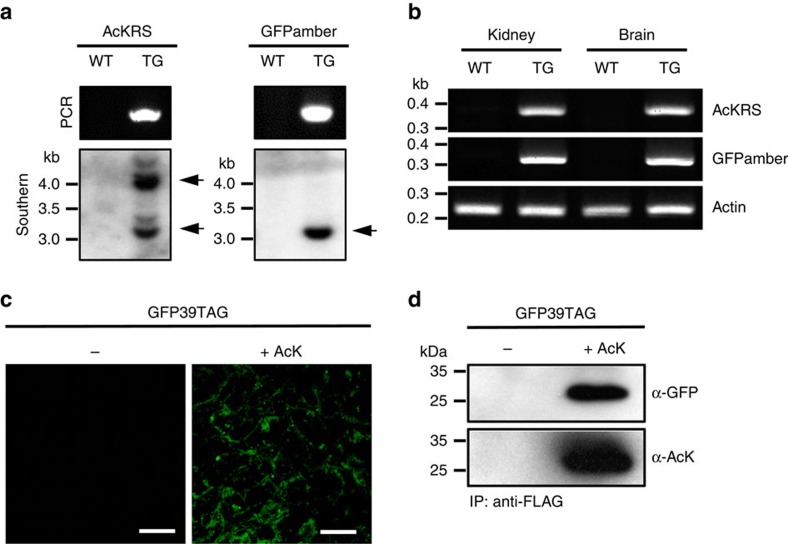
Generation of a transgenic mouse with an expanded genetic code. (**a**) Validation of the double-transgenic AcK-GFPamber mouse. PCR analysis and Southern blotting of wild type (WT) and AcK-GFPamber double-transgenic (TG) mice confirmed chromosomal integration of *AcKRS* and *GFPamber* transgenes. (**b**) Expression of *AcKRS* and *GFPamber* transgenes confirmed by RT-PCR. cDNAs were synthesized from kidney and brain total RNA and then used for PCR amplification of specific DNA fragments corresponding to *AcKRS* and *GFPamber*. Actin was used as loading control. (**c**) Fluorescence image of MEF cells established from AcK-GFPamber mouse (AcKRS/+, GFPamber/+) embryos in the presence of AcK. Scale bar, 100 μm. (**d**) Western blot analysis of anti-FLAG–immunoprecipitated proteins from MEF lysates demonstrates site-specific incorporation of AcK in response to the amber codon in MEF cells.

Next, we generated a transgenic mouse carrying the AcK-incorporation system. A linear DNA fragment containing *PylT::HA-AcKRS* was microinjected into the fertilized eggs of a C57BL/6J mouse^23^, from which the AcK transgenic mouse line was established. We also generated a transgenic mouse expressing GFPamber through chromosomal insertion of a *PylT::GFP39TAG-FLAG* transgene (GFPamber mouse). Two separate transgenic mouse strains were created to establish the stable AcK mouse and to efficiently generate a broad range of tailor-made mouse strains from it. The AcK mouse was then crossed with the GFPamber mouse to produce the double-heterozygous transgenic mouse, AcK-GFPamber. The genotype of the AcK-GFPamber mouse was confirmed by polymerase chain reaction (PCR) and Southern blot analysis (Fig. 2a and Supplementary Fig. 6). Stable chromosomal integration of *AcKRS* and *GFPamber* transgenes was further confirmed by sequencing of PCR products (Supplementary Fig. 7). Based on Southern blot analysis results, we concluded that *GFPamber* transgene is inserted into one genomic locus whereas *AcKRS* transgene is into two genomic loci (Fig. 2a). The expression of *AcKRS* and *GFPamber* transgenes in kidney and brain was confirmed by reverse transcription-PCR (RT-PCR; Fig. 2b).

### Genetic incorporation of UAAs in mouse embryonic fibroblasts

**Figure 3:**
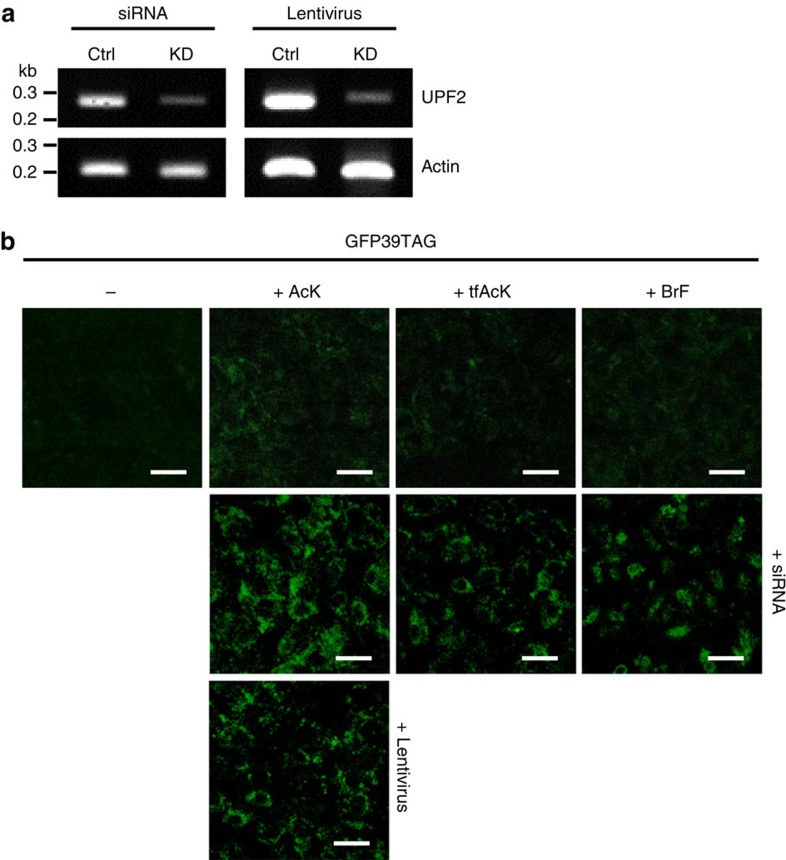
Downregulation of mouse Upf2 using siRNA and lentivirus improves amber suppression. (**a**) Knockdown of Upf2 in MEF cells by siRNA and lentivirus. RT-PCR revealed that expression of Upf2 was noticeably reduced by siRNA or lentivirus carrying shRNA against mouse Upf2. (**b**) Fluorescence image the MEF cells in the presence of UAA, (AcK, tfAcK or BrF). Downregulation of mouse Upf2 using siRNA or lentivirus clearly increased UAA-dependent GFPuv expression through enhanced efficiency in amber suppression. Scale bar, 100 μm.

Then, to demonstrate AcK incorporation, we first established primary mouse embryonic fibroblasts (MEFs) from AcK-GFPamber mouse embryos. Clear GFPuv fluorescence signals were detected in MEFs grown with AcK, but not in those grown without it (Fig. 2c). A western blot analysis of proteins isolated from MEF lysates revealed that these cells produced GFPuv only in the presence of AcK (Fig. 2d and Supplementary Fig. 8a). In addition, western blotting using an anti-AcK antibody confirmed the incorporation of AcK into GFPuv (Fig. 2d and Supplementary Fig. 8b). The AcK-dependent expression of GFPuv was also demonstrated by flow cytometric analysis (Supplementary Fig. 9). Mammalian systems have a cellular surveillance mechanism known as nonsense mediated-decay (NMD)^24^, which degrades mRNAs bearing a premature translation termination codon. Knockdown of Upf2, a major component of mammalian NMD, using small interfering RNA (siRNA) or a lentivirus carrying UPF2 shRNA (Fig. 3a), further increased AcK-dependent GFPuv expression in MEFs (Fig. 3b). Impairing NMD similarly enhanced the efficiency of amber suppression in *Caenorhabditis elegans*^9^. MEFs also exhibited tfAcK- or BrF-dependent GFPuv expression (Fig. 3b), demonstrating that site-specific UAA incorporation works well in MEFs from transgenic mice with an expanded genetic code.

### Temporal and spatial control of protein acetylation in mouse

**Figure 4:**
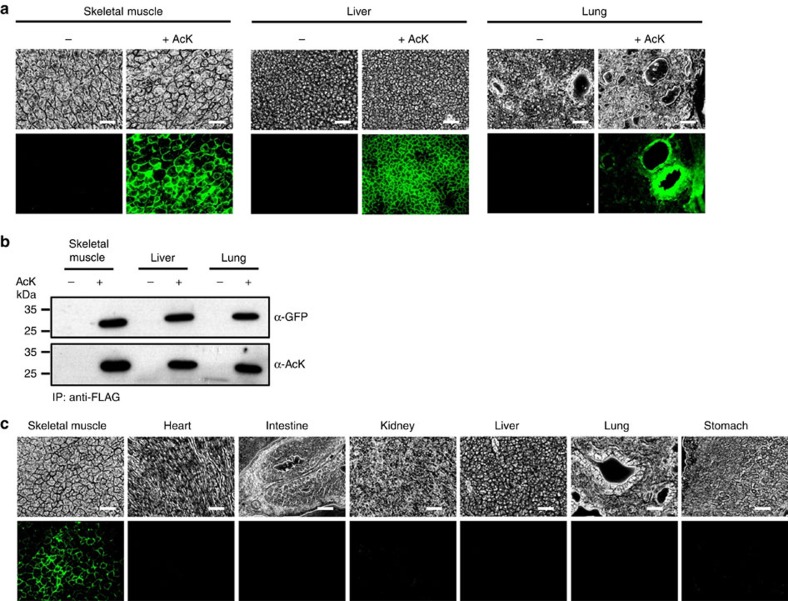
Temporal and spatial control of *in vivo* protein acetylation. (**a**) Temporal expression of acetylated GFPuv in the AcK-GFPamber mouse. The expression of GFPuv in skeletal muscle, liver and lung tissues was detected only in the AcK-injected mouse. Scale bar, 200 μm. (**b**) Western blotting of anti-FLAG–immunoprecipitated proteins from tissues of the AcK-GFPamber mouse. Acetylated GFPuv was produced after AcK injection. (**c**) Spatial expression of acetylated GFPuv in the AcK-GFPamber mouse. Acetylated GFPuv was observed only in skeletal muscle when AcK was directly delivered to the tissue. Scale bar, 200 μm.

Finally, we examined the site-specific incorporation of AcK into GFPuv in the AcK-GFPamber mouse. For the transgenic mouse to serve as an appropriate model for the physiological consequences of acetylation, it should have the ability to express acetylated proteins in a temporally and spatially controllable manner, regardless of cell type and developmental stage. Thus, to test the temporal control of protein acetylation, we started supplying AcK by daily intraperitoneal injection (50 mg in PBS) to AcK-GFPamber mice beginning at 8 weeks of age; controls received only PBS. After 5 days, we collected various tissues and prepared frozen sections for analysis of GFPuv expression. Notably, we detected clear fluorescence signals in skeletal muscle, liver and lung tissues from AcK-injected transgenic mice, but not in those from PBS-injected mice (Fig. 4a). The incorporation of AcK into GFPuv was further confirmed by western blot analysis of immunopurified GFPuv using an anti-AcK antibody (Fig. 4b and Supplementary Fig. 10). We also detected AcK-dependent GFPuv expression in other tissues (heart, intestine, kidney and stomach) from the AcK-GFPamber mouse (Supplementary Fig. 11), demonstrating ubiquitous expression of acetylated protein. The lack of GFPuv fluorescence signals in AcK-starved mice indicates that the expression of GFPuv is tightly repressed. In contrast, administration of AcK to transgenic mice quickly induced the expression of site-specifically acetylated GFPuv in various tissues. A dose-dependent fluorescence increase was observed with increasing amounts of AcK (Supplementary Fig. 12). Interestingly, some tissues such as lung and stomach show uneven expression of GFPuv, which is thought to be related to unequal delivery of AcK resulting from biased distribution of blood vessels in those tissues. Stronger fluorescence signal from bronchiole of lung and mucosa layer of stomach was found where blood vessels are more concentrated (Fig. 4a and Supplementary Fig. 11) The tight and inducible regulation of acetylated GFPuv expression demonstrates temporal control over the acetylation of a target protein in these transgenic mice. Lastly, to examine spatial control of protein acetylation, we tested tissue-specific expression of acetylated GFPuv by direct delivery of AcK to the target tissue. In mice supplied AcK directly to skeletal muscle by intramuscular injection, the expression of acetylated GFPuv was detected only in skeletal muscle, and not in other tissues (Fig. 4c). Similarly, selective expression of acetylated GFPuv in liver or kidney was also observed after direct delivery of AcK to the corresponding tissue (Supplementary Fig. 13). This local expression of acetylated protein demonstrates spatial control of acetylation of a target protein in the transgenic mouse. Precise spatial control could also be achieved using tissue-specific promoters.

Here, we have created a transgenic mouse with an expanded genetic code that enables site-specific incorporation of UAAs. This approach facilitates rapid onset of acetylation of a specific lysine residue of a target protein at any developmental stage or selected tissue of the mouse. Such temporal and spatial control of protein acetylation will be of prime importance for investigating many essential biological processes and human diseases at the tissue and organism level. This method can be easily extended to generate a wide range of custom-made transgenic mouse strains from the established AcK mouse for studying diverse proteins of interest. Furthermore, the AcKRS/tRNA^Pyl^ pair enables genetic incorporation of UAAs with diverse functionalities^21^,^22^, including deacetylase-resistant AcK analogues and UAAs enabling site-specific labelling. Hence, we also anticipate that transgenic mice with an expanded genetic code will offer a robust, versatile and powerful tool for more precisely and systematically investigating various aspects of cellular proteins.

## Methods

### Construction of plasmids

For site-specific *N*^ɛ^-acetyl-lysine (AcK) incorporation, we selected an engineered *N*^ɛ^-acetyl-lysyl-tRNA synthetase (AcKRS)/tRNA^Pyl^ pair^20^. For ubiquitous expression of AcKRS, the CMV promoter in pCDNA3 was replaced with the constitutive human EF1α promoter. Then, the gene encoding N-terminally HA-tagged AcKRS was cloned between *Kpn*I and *Not*I, generating a plasmid pAcKRS. To construct tRNA^Pyl^ expression cassette, the gene coding for tRNA^Pyl^ and the RNA polymerase III promoter U6 was synthesized (Bioneer) and the CMV immediate early region enhancer was cloned from pCDNA5 frt/TO vector (Invitrogen). DNA fragment containing tRNA^Pyl^ under the control of U6 promoter and enhancer was amplified by PCR and cloned into the plasmid pAcKRS using *Bam*HI and *Asc*I, generating a plasmid pAcKRS-tRNA. For ubiquitous expression of a model protein GFPuv, the gene encoding C-terminally FLAG-tagged GFPuv gene carrying amber stop codon at position 39 was cloned in place of the AcKRS gene in pAcKRS-tRNA, generating a plasmid pGFPamber-tRNA.

### Site-specific incorporation of UAAs in mammalian cells

Human embryonic kidney (HEK) 293 cells (Sigma-Aldrich) were grown in Dulbecco's modified Eagle's medium (DMEM) supplemented with 10% fetal bovine serum (FBS) at 37 °C with 5% CO_2_. Cells were co-transfected with 25 μg of plasmids pAckRS-tRNA and pGFPamber-tRNA using Lipofectamine 2000 (Invitrogen) at 80–90% confluence in 100-mm dishes. After 8 h of incubation, medium was replaced with fresh DMEM supplemented with 10% FBS and 10 mM UAA *N*^ɛ^-acetyl-lysine (AcK), *N*^ɛ^-trifluoroacetyl-lysine (tfAcK) or 3-bromo-phenylalanine (BrF). Cells were collected after 40 h of incubation. NIH3T3 cells (ATCC) were seeded into 100-mm dish and grown in DMEM supplemented with 10% FBS for 24 h. At ∼50% confluence, cells were transfected with 40 μg of plasmids pAckRS-tRNA and pGFPamber-tRNA using Lipofectamine 2000. After overnight incubation, medium was replaced with fresh DMEM containing 10% FBS and 10 mM UAA. Cells were collected 48 h after transfection.

### Generation of transgenic mouse strains

We created two separate transgenic mouse strains, AcKRS mouse which expresses AcKRS and tRNA^Pyl^, and GFPamber mouse which expresses GFPuv with amber stop codon at position 39 and tRNA^Pyl^. To this end, plasmids pAcKRS-tRNA and pGFPamber-tRNA were linearized with restriction enzymes *Apa*LI and *Pvu*II and then microinjected into the fertilized mouse eggs of a C57BL/6J mouse strain^23^. PCR analysis was used to identify genotype of AcKRS mouse (the forward primer, 5′-CGAAGA-CCAGACAAGCGTAAA-3′; the reverse primer, 5′-CTTGAGTCCGAATTGCTCTCTC-3′) and GFPamber mouse (the forward primer, 5′-GGTGAAGGTGATGCTACATAGG-3′; the reverse primer, 5′-TCGAGTTTGTGTCCGAGAATG-3′). Next, to generate double heterozygote transgenic mouse (AcK-GFPamber mouse), heterozygous AcK mouse (AcKRS/+) was mated to heterozygous GFPamber mouse (GFPamber/+). Genotype of double-transgenic mouse (AcKRS/+, GFPamber/+) was determined by PCR analysis and Southern blot analysis. To perform Southern blot analysis, genomic DNA of double-transgenic mouse was isolated by phenol/chloroform extraction method, digested with restriction enzymes *Bam*HI and *Sph*I, and separated on an agarose gel. Chromosome-integrated *AcKRS* an *GFPambe*r transgenes were detected with isotope labelled cDNA probes after transfer to positively charged nylon membrane. All transgenic mice were generated at the Laboratory Animal Research Center at Yonsei University. All animal experiments were performed in accordance with Korean Food and Drug Administration guidelines. Protocols were received and approved by the Institutional Animal Care and Use Committee of the Yonsei University (YLARC 2012-0087). All mice were maintained in the specific pathogen-free facility of the Yonsei Laboratory Animal Research Center.

### RT-PCR analysis of *AcKRS* and *GFPamber* transgenes

To examine the expression of *AcKRS* and *GFPamber* in different mouse tissues, RNA was isolated from brain and kidney. A 305 bp cDNA fragment was amplified from RNA by using primers binding to cDNA of AcKRS (the forward primer, 5′-CGCGGAAGAAAGGGAGAATTA-3′; the reverse primer, 5′-CTTTGCCGTCGGACTCTTT-3′) and a 329 bp cDNA fragment was amplified from RNA by using primers binding to cDNA of GFPamber (the forward primer, 5′-GGTGAAGGTGATGCTACATAGG-3′; the reverse primer, 5′-TCGAGTTTGTGTCCGAGAATG-3′). Expression of *actin* was determined by RT-PCR using specific primers (the forward primer, 5′-GTGACGTTGACATCCGTAAAGA-3′; the reverse primer, 5′-GCCGGACTCATCGTACTCC-3′).

### Treatment of transgenic mouse

Animals were housed under a 12-h light/dark cycle in standard animal cages and were provided with food and water *ad libitum*. To induce expression of acetylated GFP, 50 mg of AcK (Sigma) dissolved in PBS was intraperitoneally injected to four 8-week old double-transgenic mice (AcKRS/+, GFPamber/+) on a daily basis. For control experiment, two 8-week old double-transgenic mice were injected with PBS. After 5 days of AcK injection, double-transgenic mice were killed and tissues were collected. For tissue-specific induction of acetylated GFP expression, 50 mg of AcK was injected directly to target tissues. In this study, a mixture of male and female animals were used.

### Fluorescence microscopic analysis of mouse tissues

Immediately after killing, tissues were collected from double-transgenic mouse (AcKRS/+, GFPamber/+), embedded in Tissue-Tek O.C.T. (Sakura Finetek) and stored in −80 °C freezer. Following cryo-sectioning of the tissue blocks, frozen sections with a thickness of 20 μm were prepared with a cryostat and mounted on glass slides. GFPuv fluorescence was detected using a fluorescence microscope (Axiovert 200FL, Zeiss) and the images were captured and digitalized using the associated software, Axiovision.

### Mouse embryonic fibroblast

Primary MEF cells were established from double-transgenic mouse (AcKRS/+, GFPamber/+) embryos at 13.5 dpc^25^.

Briefly, whole embryos were collected from pregnant females and minced with scalpel blades. Embryos were then incubated with 0.05% trypsin at 37 °C for 15 min and plated in DMEM (Life Technologies) supplemented with 10% FBS (Sigma). Genotype of embryos were identified with DNA isolated from established MEF cells. For induction of acetylated GFP expression, 10 mM AcK was added to culture medium. The expression of acetylated GFP was visualized using fluorescence microscopy 24 h after induction.

### Downregulation of mouse Upf2 using siRNA

Knockdown of Upf2 in MEF cells was performed as follows^26^. Briefly, MEF cells (2 × 10^6^) established from double-transgenic mouse (AcKRS/+, GFPamber/+) were grown in DMEM media containing 10% FBS in 60-mm dishes and transiently transfected with 100 nM of siRNA against Upf2 (5′-UUUAGGUUGAUUAACCUCCAUUCCC-3′) using lipofectamine (Invitrogen). siRNA against mouse Upf2 and control siRNA were purchased from Bioneer.

### Lentivirus-mediated downregulation of mouse Upf2

Mouse shRNA against mouse Upf2 (5′-TTTAGGTTGATTAACCTCCATTCCC-3′) was cloned into lentiviral vector, pLKO.1-TRC (Addgene), generating a plasmid pLKO.1-Upf2. To produce lentiviral particle, pLKO.1-Upf2 was transfected to 293TN cells (System Biosciences) together with plasmids pGag-pol and pVSV-G with Lipofectamine^27^. 48 h after transfection, viral particles were collected and infected into MEF cells. Infected MEF cells were selected by 1 μg per ml puromycin for a week.

### Immunoprecipitation and western blot analysis

To extract proteins, ∼100 mg of cells and tissue samples were homogenized in a lysis buffer (50 mM Tris-HCl [pH 7.4], 150 mM NaCl, 2 mM EDTA, 1%(v/v) Triton X-100, 0.1% NP-40, protease inhibitor cocktail) using bead homogenizer (MP Biomedicals). After sonication using a Bioruptor UCD-200 (Diagenode), the resulting lysates were centrifuged at 12,000 × g for 5 min at 4 °C. The supernatant was collected and used for immunoprecipitation. Immunoprecipitation of GFP was performed^28^. Briefly, the samples were mixed with 20 μl of anti-FLAG magnetic bead (Sigma) and incubated for 12 h at 4 °C in a rotary shaker. After washing the beads with ice cold washing buffer (50 mM Tris-HCl, 150 mM NaCl, pH 7.4) twice, GFP was eluted using elution buffer (0.1 M Glycine-HCl, pH 3.0). Eluates were immediately neutralized using 1.0 M Tris-HCl (pH 8.0). Western blot analysis was performed according to standard procedure using anti-GFP (Abcam, Cat. No. ab6556, at 1:1,000 dilution), anti-FLAG (Abcam, Cat. No. ab1257, at 1:1,000 dilution) and anti-acetyl-lysine (BioLegend, Cat. No. 623402, at 1:200 dilution) antibodies.

### Mass analysis

Protein samples were electrophoresed on 15% SDS-PAGE gel, and the protein band was excised from the gel. In-gel tryptic digestion was carried out^29^. Briefly, the excised band was cut into small pieces and de-stained with 100 mM ammonium bicarbonate/acetonitrile (1:1, v/v) for 30 min. Then, the gel pieces were mixed with 500 μl of neat acetonitrol and incubated at room temperature until they became while and shrunk. After removing acetonitrile solution, the gel pieces were completely covered with a solution of 10 ng per μl trypsin (10 mM ammonium bicarbonate, 10% acetonitrile (v/v)) and incubated on ice for 2 h. Then, the sample tube was placed in the incubator at 37 °C overnight for complete digestion. 0.5 μl of trypsin-digested sample was mixed with 1.5 μl of matrix solution (20 mg per ml 2,5-dihydroxybenzoic acid (DHB) dissolved in 0.1% TFA in acetonitrile/water, 1:1). An aliquot of 1 μl of the mixture was spotted on a ground steel MTP384 for analysis. The mass data were acquired on a bruker autoflex III MALDI-TOF mass spectrometer in reflectron mode.

### Data availability

All relevant data are available from the authors.

## Additional information

**How to cite this article:** Han, S. *et al*. Expanding the genetic code of *Mus musculus*. *Nat. Commun.* **8**, 14568 doi: 10.1038/ncomms14568 (2017).

**Publisher's note:** Springer Nature remains neutral with regard to jurisdictional claims in published maps and institutional affiliations.

## Supplementary Material

Supplementary Information Supplementary Figures and Supplementary Table
