# Supplementary material for: Expanding the genetic code of Mus musculus
Source: Nat Commun. 2017 Feb 21;8:14568. doi: 10.1038/ncomms14568 (PMC5321798; doi:10.1038/ncomms14568)
Supplement: Supplementary Information — Supplementary Figures and Supplementary Table [file ncomms14568-s1.pdf]

gacggatcgggagatctcccgatcccctatggtcgactctcagtacaatctgctctgatgccgcatagttaagccagtatctgctccctgcttgtgtgttggcgcgccaaaaaccgcacttgt
ccggaaaccccgggaatctaacccggctgaacggatttagagtccattcgatctacatgatcaggtttccctcgagtctagaagatctcggtgtttcgtcctttccacaagatatataaagcc
aagaaatcgaaatactttcaagttacggtaagcatatgatagtccattttaaaacataattttaaaactgcaaactacccaagaaattattactttctacgtcacgtattttgtactaatatctttgt
gtttacagtcaaattaattctaattatctctctaacagccttgtatcgtatatgcaaatatgaaggaatcatgggaaataggccctcttcctgcccagatctgtcccgttgattttggtgccaaaa
caaactcccattgacgtcaatggggtggagacttggaaatccccgtgagtcaaaccgctatccacgcccattgatgtactgccaaaaccgcatcaccatggtaatagcgatgactaatacgt
agatgtactgccaagtaggaaagtcccataaggtcatgtactgggcataatgccaggcgggccatttaccgtcattgacgtcaatagggggcgtacttggcatatgatacacttgatgtac
tgccaagtgggcagtttaccgtaaatactccacccattgacgtcaatggaaagtccctattggcgttactatgggaacatacgtcattattgacgtcaatgggcgggggtcgttgggcggt
cagccaggcgggccatttaccgtaagttatgtaacgcggaactccatatatgggctatgaactaatgaccccgtaattgattactattaataactaggatccaagctacaacaaggcaaggc
ttgaccgagaattccgtgaggctccggtgcccgtcagtgggcagagcgcacatcgcccacagtccccgagaagttgggggggaggggtcggcaattgaaccggtgcctagagaaggtg
gcgcggggtaaactgggaaagtgatgtcgtgtactggctccgcctttttcccgagggtgggggagaaccgtatataagtgcagtagtcgccgtgaacgttctttttcgcaacgggtttgc
cgccagaacacaggtaagtgccgtgtgtggttcccgcgggcctggcctctttacgggttatggcccttgcgtgccttgaattacttccacctggctccagtacgtgattcttgatcccgagct
ggagccaggggcgggccttgcgctttaggagccccttcgcctcgtgcttgagttgaggcctggcctgggcgctggggccgccgcgtgcgaatctggtggcaccttcgcgcctgtctcgc
tgctttcgataagtctctagccatttaaaatttttgatgacctgctgcgacgctttttttctggcaagatagtcttgtaaatgcgggccaggatctgcacactggtatttcggtttttggggccg
cgggcggcgacggggcccgtgcgtcccagcgcacatgttcggcgaggcggggcctgcgagcgcggccaccgagaatcggacgggggtagtctcaagctggccggcctgctctggt
gcctggcctcgcgccgccgtgtatcgccccgccctgggcggcaaggctggcccggtcggcaccagttgcgtgagcggaaagatggccgcttcccggccctgctccagggggctcaaaa
tggaggacgcggcgctcgggagagcgggcgggtgagtcacccacacaaaggaaaggggcctttccgtcctcagccgtcgcttcatgtgactccacggagtaccgggcgccgtccagg
cacctcgattagttctggagcttttggagtacgtcgtctttaggttgggggggaggggttttatgcgatggagtttccccacactgagtgggtggagactgaagttaggccagcttggcact
tgatgtaattctccttggaatttgccctttttgagtttggatcttggttcattctcaagcctcagacagtggttcaaagtttttttcttccatttcaggtgtcgtgaacaccgggggtaccgccacc
atgtatccatatgatgttccagattatgctatggataaaaaaccactaaacactctgatatccgcaaccgggctctggatgtccaggaccggaacaattcataaaataaaacaccacgaagtc
tctcgaagcaaaatctatattgaaatggcatgcggaggccaccttgttgtaaacaactccaggagcagcaggactgcaagagcgctcaggcaccacaaatacaggaagacctgcaaacg
ctgcagggtttcggatgaggatctcaataagttcctcacaaaggcaaacgaagaccagacaagcgtaaaagtcaaggtcgtttctgcccctaccagaacgaaaaaggcaatgccaaaatc
cgttgcgagagccccgaaacctcttgagaatacagaagcggcacaggctcaaccttctggatctaaattttcacctgcgataccggtttccacccaagagtcagtttctgtcccggcatctgt
ttcaacatcaatatcaagcatttctacaggagcaactgcatccgcactggtaaaagggaatacgaatcccattacatccatgtctgcccctgttcaggcaagtgcccccgcacttacgaagaa
ccagactgacaggcttgaagtcctgttaaacccaaaagatgagatttccctgaattccggcaagcctttcagggagcttgagtccgaattgctctctcgcagaaaaaaagacctgcagcag
atctacgcggaagaaagggagaattatctggggaaactcgagcgtgaaattaccaggttctttgtggacaggggttttctggaaataaaatccccgatcctgatccctcttgagtatatcg
aaaggatgggcattgataatgataccgaactttcaaaacagatcttcagggttgacaagaacttctgcctgagacccatgatggctccaaaccttctgaactacgcgcgcaagcttgacag
ggccctgcctgatccaataaaaatttttgaaataggcccatgctacagaaaagagtccgacggcaaagaacacctcgaagagtttaccatgctgaacttcttccagatgggatcgggatgc
acacgggaaaatcttgaaagcataattacggacttcctgaaccacctgggaattgatttcaagatcgtaggcgattcctgcatggtctatggggataccctttgatgtaatgcacggagacct
ggaactttcctctgcagtagtcggacccataccgcttgaccgggaatggggtattgataaaccctggataggggcaggtttcgggctcgaacgccttctaaaggttaaacacgactttaaa
aatatcaagagagctgcaaggtccgagtcttactataacgggatttctaccaacctgtgagcggccgctcgagcatgcatctagagggccctattctatagtgtcacctaaatgctagagct
cgctgatcagcctcgactgtgccttctagttgccagccatctgttgtttgcccctcccccgtgccttccttgaccctggaaggtgccactcccactgtcctttcctaataaaatgaggaaattgca
tcgcattgtctgagtaggtgtcattctattctggggggtggggtggggcaggacagcaagggggaggattgggaagacaatagcaggcatgctggggatgcggtgggctctatggc
ttctgaggcggaaagaaccagctggggctctagggggtatccccacgcgccctgtagcggcgcattaagcgcggcgggtgtggtggttacgcgcagcgtgaccgctacacttgccagc
gccctagcgcccgctcctttcgctttcttcccttcctttctcgccacgttcgccggctttccccgtcaagctctaaatcgggggcatccctttagggttccgatttagtgctttacggcacctcgacc
ccaaaaaacttgattagggtgatggttcacgtagtgggccatcgccctgatagacggtttttcgccctttgacgttggagtccacgttctttaatagtggactcttgttccaaactggaacaac
actcaaccctatctcggtctattcttttgatttataagggattttggggatttcggcctattggttaaaaaatgagctgatttaacaaaaatttaacgcgaattaattctgtggaatgtgtgtcag
ttagggtgtggaaagtccccaggctccccaggcaggcagaagtatgcaaagcatgcatctcaattagtcagcaaccaggtgtggaaagtccccaggctccccagcaggcagaagtatgc
aaagcatgcatctcaattagtcagcaaccatagtcccgcccctaactccgcccatcccgcccctaactccgcccagttccgcccattctccgccccatggctgactaattttttttatttatgcaga
ggccgaggccgcctctgcctctgagctattccagaagtagtgaggaggcttttttggaggcctaggcttttgcaaaaagctcccgggagcttgtatatccattttcggatctgatcaagaga
caggatgaggatcgtttcgcatgattgaacaagatggattgcacgcaggttctccggccgcttgggtggagaggctattcggctatgactgggcacaacagacaatcggctgctctgat
gccgccgtgttccggctgtcagcgcaggggcgcccggttctttttgtcaagaccgacctgtccggtgccctgaatgaactgcaggacgaggcagcgcggctatcgtggctggccacgac
gggcgttccttgcgcagctgtgctcgacgttgtcactgaagcgggaagggactggctgctattgggcgaagtgccggggcaggatctcctgtcatctcaccttgctcctgccgagaaagt
atccatcatggctgatgcaatgcggcggctgcatacgcttgatccggctacctgcccattcgaccaccaagcgaaacatcgcatcgagcgagcacgtactcggatggaagccggtcttgtc
gatcaggatgatctggacgaagagcatcaggggctcgcgccagccgaactgttcgccaggctcaaggcgcgcatgcccgacggcgaggatctcgtcgtgacccatggcgatgcctgct
tgccgaatatcatggtggaaaatggccgcttttctggattcatcgactgtggccggctgggtgtggcggaccgctatcaggacatagcgttggctacccgtgatattgctgaagagcttg
gcggcgaatgggctgaccgcttcctcgtgctttacggtatcgccgctcccgattcgcagcgcatcgccttctatcgccttcttgacgagttcttctgagcgggactctggggttcgaaatgac
cgaccaagcgacgcccaacctgccatcacgagatttcgattccaccgccgccttctatgaaaggttgggcttcggaatcgttttccgggacgccggctggatgatcctccagcgcggggat
ctcatgctggagttcttcgcccaccccaacttgtttattgcagcttataatggttacaaataaagcaatagcatcacaaatttcacaaataaagcatttttttcactgcattctagttgtggtttgt
ccaaactcatcaatgtatcttatcatgtctgtataccgtcgacctctagctagagcttggcgtaatcatggtcatagctgtttcctgtgtgaaattgttatccgctcacaattccacacaacatac
gagccggaagcataaagtgtaaagcctggggtgcctaatgagtgagctaactcacattaattgcgttgcgctcactgcccgctttccagtcgggaaacctgtcgtgccagctgcattaatg
aatcggccaacgcgcggggagaggcggtttgcgtattgggcgctcttccgcttcctcgctcactgactcgctgcgctcggtcgttcggctgcggcgagcggtatcagctcactcaaaggc
ggtaatacggttatccacagaatcaggggataacgcaggaaagaacatgtgagcaaaaggccagcaaaaggccaggaaccgtaaaaaggccgcgttgctggcgtttttccataggctc
cgcccccctgacgagcatcacaaaaatcgacgctcaagtcagaggtggcgaaacccgacaggactataaagataccaggcgtttccccctggaagctccctcgtgcgctctcctgttccga
ccctgccgcttaccggatacctgtccgcctttctcccttcgggaagcgtggcgctttctcaatgctcacgctgtaggtatctcagttcggtgtaggtcgttcgctccaagctgggctgtgtgca
cgaaccccccgttcagcccgaccgctgcgccttatccggtaactatcgtcttgagtccaacccggtaagacacgacttatcgccactggcagcagccactggtaacaggattagcagagcg
aggtatgtaggcggtgctacagagttcttgaagtggtggcctaactacggctacactagaaggacagtatttggtatctgcgctctgctgaagccagttaccttcggaaaaagagttggta
gctcttgatccggcaaacaaaccaccgctggtagcggtggtttttttgtttgcaagcagcagattacgcgcagaaaaaaaggatctcaagaagatcctttgatcttttctacggggtctgac
gctcagtggaacgaaaactcacgttaagggattttggtcatgagattatcaaaaaggatcttcacctagatccttttaaattaaaaatgaagttttaaatcaatctaaagtatatatgagtaaa
cttggtctgacagttaccaatgcttaatcagtgaggcacctatctcagcgatctgtctatttcgttcatccatagttgcctgactccccgtcgtgtagataactacgatacgggagggcttacca
tctggccccagtgctgcaatgataccgcgagacccacgctcaccggctccagatttatcagcaataaaccagccagccggaagggccgagcgcagaagtggtcctgcaactttatccgcct
ccatccagtctattaattgttgccgggaagctagagtaagtagttcgccagttaatagtttgcgcaacgttgttgccattgctacaggcatcgtggtgtcacgctcgtcgtttggtatggcttc
attcagctccggttcccaacgatcaaggcgagttacatgatcccccatgttgtgcaaaaaagcggttagctccttcggtcctccgatcgttgtcagaagtaagttggccgcagtgttatcact
catggttatggcagcactgcataattctcttactgtcatgccatccgtaagatgcttttctgtgactggtgagtactcaaccaagtcattctgagaatagtgtatgcggcgaccgagttgctct
tgcccggcgtcaatacgggataataccgcgccacatagcagaactttaaaagtgctcatcattggaaaacgttcttcggggcgaaaactctcaaggatcttaccgctgttgagatccagttc
gatgtaacccactcgtgcacccaactgatcttcagcatcttttactttcaccagcgtttctgggtgagcaaaaacaggaaggcaaaatgccgcaaaaaagggaataagggcgacacggaa
atgttgaatactcatactcttcctttttcaatattattgaagcatttatcagggttattgtctcatgagcggatacatatttgaatgtatttagaaaaataaacaaataggggttccgcgcacattt
ccccgaaaagtgccacctgacgtc

Supplementary Figure 1. Sequence of pAcKR-tRNA plasmid.

gacggatcgggagatctcccgatcccctatggtcgactctcagtacaatctgctctgatgccgcatagttaagccagtatctgctccctgcttgtgtgttggcgcgccaaaaaccgca
cttgtccggaaaccccgggaatctaacccggctgaacggatttagagtccattcgatctacatgatcaggtttccctcgagtctagaagatctcggtgtttcgtcctttccacaagata
tataaagccaagaaatcgaaatactttcaagttacggtaagcatatgatagtccattttaaaacataattttaaaactgcaaactacccaagaaattattactttctacgtcacgtatttt
gtactaatatctttgtgtttacagtcaaattaattctaattatctctctaacagccttgtatcgtatatgcaaatatgaaggaatcatgggaaataggccctcttcctgcccagatctgtcc
cgttgattttggtgccaaaacaaactcccattgacgtcaatggggtggagacttggaaatccccgtgagtcaaaccgctatccacgcccattgatgtactgccaaaaccgcatcacc
atggtaatagcgatgactaatacgtagatgtactgccaagtaggaaagtcccataaggtcatgtactgggcataatgccaggcgggccatttaccgtcattgacgtcaatagggg
gcgtacttggcatatgatacacttgatgtactgccaagtgggcagtttaccgtaaatactccacccattgacgtcaatggaaagtccctattggcgttactatgggaacatacgtcatt
attgacgtcaatgggcgggggtcgttgggcggtcagccaggcgggccatttaccgtaagttatgtaacgcggaactccatatatgggctatgaactaatgaccccgtaattgatta
ctattaataactaggatccaagctacaacaaggcaaggcttgaccgagaattccgtgaggctccggtgcccgtcagtgggcagagcgcacatcgcccacagtccccgagaagttg
gggggaggggtcggcaattgaaccggtgcctagagaaggtggcgcggggtaaactgggaaagtgatgtcgtgtactggctccgcctttttcccgagggtgggggagaaccg
tatataagtgcagtagtcgccgtgaacgttctttttcgcaacgggtttgccgccagaacacaggtaagtgccgtgtgtggttcccgcgggcctggcctctttacgggttatggccctt
gcgtgccttgaattacttccacctggctccagtacgtgattcttgatcccgagctggagccaggggcgggccttgcgctttaggagccccttcgcctcgtgcttgagttgaggcctg
gcctgggcgctggggccgccgcgtgcgaatctggtggcaccttcgcgcctgtctcgctgctttcgataagtctctagccatttaaaatttttgatgacctgctgcgacgcttttttct
ggcaagatagtcttgtaaatgcgggccaggatctgcacactggtatttcggtttttggggccgcgggcggcgacggggcccgtgcgtcccagcgcacatgttcggcgaggcgg
ggcctgcgagcgcggccaccgagaatcggacgggggtagtctcaagctggccggcctgctctggtgcctggcctcgcgccgccgtgtatcgccccgccctgggcggcaaggct
ggcccggtcggcaccagttgcgtgagcggaaagatggccgcttcccggccctgctccagggggctcaaaatggaggacgcggcgctcgggagagcgggcgggtgagtcacc
cacacaaaggaaaggggcctttccgtcctcagccgtcgcttcatgtgactccacggagtaccgggcgccgtccaggcacctcgattagttctggagcttttggagtacgtcgtcttt
aggttggggggaggggttttatgcgatggagtttccccacactgagtgggtggagactgaagttaggccagcttggcacttgatgtaattctccttggaatttgccctttttgagtt
tggatcttggttcattctcaagcctcagacagtggttcaaagtttttttcttccatttcaggtgtcgtgaacaccgggggtaccgccaccatggaaagtaaaggagaagaacttttcac
tggagttgtcccaattcttgttgaattagatggtgatgttaatgggcacaaattttctgtcagtggagagggtgaaggtgatgcaacatagggaaaacttacccttaaatttatttgc
actactggaaaactacctgttccgtggccaacacttgtcactactttctcttatggtgttcaatgcttttcccgttatccggatcatatgaaacggcatgactttttcaagagtgccatgcc
cgaaggttatgtacaggaacgcactatatctttcaaagatgacgggaactacaagacgcgtgctgaagtcaagtttgaaggtgatacccttgttaatcgtatcgagttaaaaggtat
tgattttaaagaagatggaaacattctcggacacaaactcgagtacaactataactcacacaatgtatacatcacggcagacaaacaaaagaatggaatcaaagctaacttcaaaat
tcgccacaacattgaagatggttccgttcaactagcagaccattatcaacaaaatactccaattggcgatggccctgtccttttaccagacaaccattacctgtcgacacaatctgccct
ttcgaaagatcccaacgaaaagcgtgaccacatggtccttcttgagtttgtaactgctgctgggattacacatggcatggatgagctctacaaagactacaaggacgacgatgaca
agtgagcggccgctcgagcatgcatctagagggccctattctatagtgtcacctaaatgctagagctcgctgatcagcctcgactgtgccttctagttgccagccatctgttgtttgcc
cctcccccgtgccttccttgaccctggaaggtgccactcccactgtcctttcctaataaaatgaggaaattgcatcgcattgtctgagtaggtgtcattctattctggggggtggggtg
gggcaggacagcaagggggaggattgggaagacaatagcaggcatgctggggatgcggtgggctctatggcttctgaggcggaaagaaccagctggggctctagggggt
atccccacgcgccctgtagcggcgcattaagcgcggcgggtgtggtggttacgcgcagcgtgaccgctacacttgccagcgccctagcgcccgctcctttcgctttcttcccttcctt
tctcgccacgttcgccggctttccccgtcaagctctaaatcgggggcatccctttagggttccgatttagtgctttacggcacctcgaccccaaaaaacttgattagggtgatggttcac
gtagtgggccatcgccctgatagacggtttttcgccctttgacgttggagtccacgttctttaatagtggactcttgttccaaactggaacaacactcaaccctatctcggtctattcttt
tgatttataagggattttggggatttcggcctattggttaaaaaatgagctgatttaacaaaaatttaacgcgaattaattctgtggaatgtgtgtcagttagggtgtggaaagtccc
caggctccccaggcaggcagaagtatgcaaagcatgcatctcaattagtcagcaaccaggtgtggaaagtccccaggctccccagcaggcagaagtatgcaaagcatgcatctca
attagtcagcaaccatagtcccgcccctaactccgcccatcccgcccctaactccgcccagttccgcccattctccgccccatggctgactaattttttttatttatgcagaggccgaggc
cgcctctgcctctgagctattccagaagtagtgaggaggcttttttggaggcctaggcttttgcaaaaagctcccgggagcttgtatatccattttcggatctgatcaagagacagg
atgaggatcgtttcgcatgattgaacaagatggattgcacgcaggttctccggccgcttgggtggagaggctattcggctatgactgggcacaacagacaatcggctgctctgat
gccgccgtgttccggctgtcagcgcaggggcgcccggttctttttgtcaagaccgacctgtccggtgccctgaatgaactgcaggacgaggcagcgcggctatcgtggctggcc
acgacgggcgttccttgcgcagctgtgctcgacgttgtcactgaagcgggaagggactggctgctattgggcgaagtgccggggcaggatctcctgtcatctcaccttgctcctg
ccgagaaagtatccatcatggctgatgcaatgcggcggctgcatacgcttgatccggctacctgcccattcgaccaccaagcgaaacatcgcatcgagcgagcacgtactcggatg
gaagccggtcttgtcgatcaggatgatctggacgaagagcatcaggggctcgcgccagccgaactgttcgccaggctcaaggcgcgcatgcccgacggcgaggatctcgtcgt
gacccatggcgatgcctgcttgccgaatatcatggtggaaaatggccgcttttctggattcatcgactgtggccggctgggtgtggcggaccgctatcaggacatagcgttggcta
cccgtgatattgctgaagagcttggcggcgaatgggctgaccgcttcctcgtgctttacggtatcgccgctcccgattcgcagcgcatcgccttctatcgccttcttgacgagttcttc
tgagcgggactctggggttcgaaatgaccgaccaagcgacgcccaacctgccatcacgagatttcgattccaccgccgccttctatgaaaggttgggcttcggaatcgttttccgg
gacgccggctggatgatcctccagcgcggggatctcatgctggagttcttcgcccaccccaacttgtttattgcagcttataatggttacaaataaagcaatagcatcacaaatttcac
aaataaagcatttttttcactgcattctagttgtggtttgtccaaactcatcaatgtatcttatcatgtctgtataccgtcgacctctagctagagcttggcgtaatcatggtcatagctgt
ttcctgtgtgaaattgttatccgctcacaattccacacaacatacgagccggaagcataaagtgtaaagcctggggtgcctaatgagtgagctaactcacattaattgcgttgcgctc
actgcccgctttccagtcgggaaacctgtcgtgccagctgcattaatgaatcggccaacgcgcggggagaggcggtttgcgtattgggcgctcttccgcttcctcgctcactgactc
gctgcgctcggtcgttcggctgcggcgagcggtatcagctcactcaaaggcggtaatacggttatccacagaatcaggggataacgcaggaaagaacatgtgagcaaaaggcc
agcaaaaggccaggaaccgtaaaaaggccgcgttgctggcgtttttccataggctccgcccccctgacgagcatcacaaaaatcgacgctcaagtcagaggtggcgaaacccga
caggactataaagataccaggcgtttccccctggaagctccctcgtgcgctctcctgttccgaccctgccgcttaccggatacctgtccgcctttctcccttcgggaagcgtggcgctt
tctcaatgctcacgctgtaggtatctcagttcggtgtaggtcgttcgctccaagctgggctgtgtgcacgaaccccccgttcagcccgaccgctgcgccttatccggtaactatcgtct
tgagtccaacccggtaagacacgacttatcgccactggcagcagccactggtaacaggattagcagagcgaggtatgtaggcggtgctacagagttcttgaagtggtggcctaa
ctacggctacactagaaggacagtatttggtatctgcgctctgctgaagccagttaccttcggaaaaagagttggtagctcttgatccggcaaacaaaccaccgctggtagcggtg
gtttttttgtttgcaagcagcagattacgcgcagaaaaaaaggatctcaagaagatcctttgatcttttctacggggtctgacgctcagtggaacgaaaactcacgttaagggatttt
ggtcatgagattatcaaaaaggatcttcacctagatccttttaaattaaaaatgaagttttaaatcaatctaaagtatatatgagtaaacttggtctgacagttaccaatgcttaatcagt
gaggcacctatctcagcgatctgtctatttcgttcatccatagttgcctgactccccgtcgtgtagataactacgatacgggagggcttaccatctggccccagtgctgcaatgatacc
gcgagacccacgctcaccggctccagatttatcagcaataaaccagccagccggaagggccgagcgcagaagtggtcctgcaactttatccgcctccatccagtctattaattgttg
ccgggaagctagagtaagtagttcgccagttaatagtttgcgcaacgttgttgccattgctacaggcatcgtggtgtcacgctcgtcgtttggtatggcttcattcagctccggttcc
caacgatcaaggcgagttacatgatcccccatgttgtgcaaaaaagcggttagctccttcggtcctccgatcgttgtcagaagtaagttggccgcagtgttatcactcatggttatgg
cagcactgcataattctcttactgtcatgccatccgtaagatgcttttctgtgactggtgagtactcaaccaagtcattctgagaatagtgtatgcggcgaccgagttgctcttgcccg
gcgtcaatacgggataataccgcgccacatagcagaactttaaaagtgctcatcattggaaaacgttcttcggggcgaaaactctcaaggatcttaccgctgttgagatccagttcg
atgtaacccactcgtgcacccaactgatcttcagcatcttttactttcaccagcgtttctgggtgagcaaaaacaggaaggcaaaatgccgcaaaaaagggaataagggcgacacg
gaaatgttgaatactcatactcttcctttttcaatattattgaagcatttatcagggttattgtctcatgagcggatacatatttgaatgtatttagaaaaataaacaaataggggttccg
cgcacatttccccgaaaagtgccacctgacgtc

Supplementary Figure 2. Sequence of pGFPamber-tRNA plasmid.

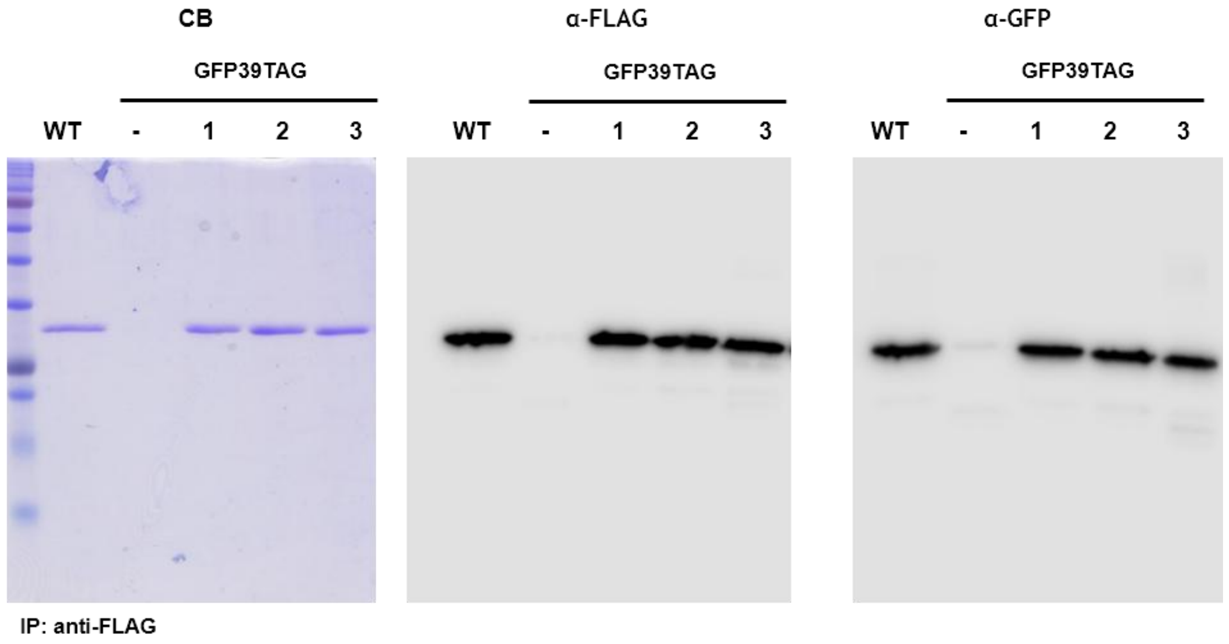

Supplementary Figure 3. Uncropped Western blot images for Figure 1d.

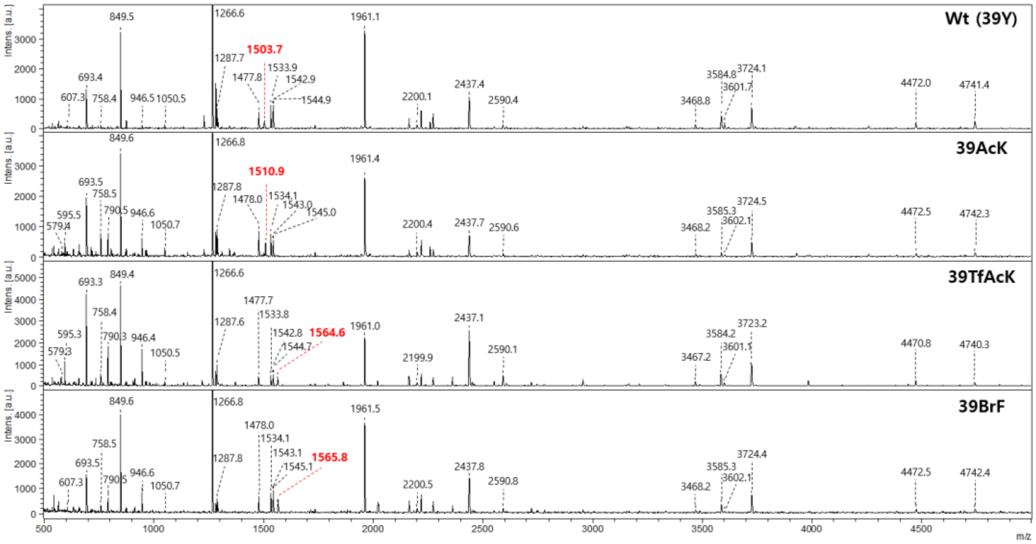

Residues	Peptide sequence	Expected mass $[M+H]^+$	Measured mass $[M+H]^+$			
			Wt(39Y)	39AcK	39TfAcK	39BrF
1-4	MASK	436.2	-	-	-	-
5-27	GEELFTGVVPILVELDGDVNGHK	2437.3	2437.4	2437.7	2437.1	2437.8
**28-42**	**FSVSGEGEGDATYGK**	**1503.7**	**1503.7**	**1510.9**	**1564.6**	**1565.8**
43-74	LTLKFICTTGKLPVPWPTLVTTFSYGVQCFSR	3603.9	3601.7	3602.1	3601.1	3602.1
75-80	YPDHMK	790.4	ND	790.5	790.3	790.5
75-81	YPDHMKR	946.5	946.5	946.6	946.4	946.6
81-86	RHDFFK	849.4	849.5	849.6	849.4	849.6
82-86	HDFFK	693.3	693.4	693.5	693.3	693.5
87-97	SAMPEGYVQER	1266.6	1266.6	1266.8	1266.6	1266.8
98-102	TISFK	595.3	ND	595.5	595.3	ND
98-108	TISFKDDGNYK	1287.6	1287.7	1287.8	1287.6	1287.8
98-110	TISFKDDGNYKTR	1544.8	1544.9	1545.0	1544.7	1545.1
111-123	AEVKFEGDTLVNR	1477.8	1477.8	1478.0	1477.7	1478.0
111-127	AEVKFEGDTLVNRIELK	1961.1	1961.1	1961.4	1961.0	1961.5
115-123	FEGDTLVNR	1050.5	1050.5	1050.7	1050.5	1050.7
115-127	FEGDTLVNRIELK	1533.8	1533.9	1534.1	1533.8	1534.1
128-132	GIDFK	579.3	ND	579.4	579.3	ND
128-141	GIDFKEDGNILGHK	1542.8	1542.9	1543.0	1542.8	1543.1
128-157	GIDFKEDGNILGHKLEYNYN SHNVYITADK	3467.7	3468.8	3468.2	3467.2	3468.2
128-159	GIDFKEDGNILGHKLEYNYNSHNVYITADKQK	3723.8	3724.1	3724.5	3723.2	3724.4
142-159	LEYNYNSHNVYITADKQK	2200.1	2200.1	2200.4	2199.9	2200.5
160-163	NGIK	431.3	-	-	-	-
164-167	ANFK	479.3	-	-	-	-
168-210	IRHNIEDGSVQLADHYQQNT PIGDGPVLLPDNHYLSTQSA LSK	4741.4	4741.4	4742.3	4740.3	4742.4
170-210	IRHNIEDGSVQLADHYQQNTPIGDGPVLLPDNHYLSTQSALSK	4472.2	4472.0	4472.5	4470.8	4472.5
211-215	DPNEK	602.3	ND	ND	ND	ND
211-216	DPNEKR	758.4	758.4	758.5	758.4	758.5
216-216	R	175.1	-	-	-	-
217-239	DHMVLLEFVTAAGITHGMDE LYK	2590.3	2590.4	2590.6	2590.1	2590.8
217-247	DHMVLLEFVTAAGITHGMDELYKDYKDDDDK	3584.7	3584.8	3585.3	3584.2	3585.3
243-247	DDDDK	607.2	607.3	ND	ND	607.3

Supplementary Figure 4. Mass spectrum of in-gel trypsin digestion of GFPuv wild-type (Wt) and mutants carrying UAAs at position 39 (39AcK, 39TfAcK, and 39BrF). Each peak in the spectrum (top) represents a tryptic peptide (bottom). Mass spectra were identical among GFPuv samples except peptide fragment (28-42) carrying UAAs. The tryptic peptide carrying UAAs and corresponding peaks are indicated.

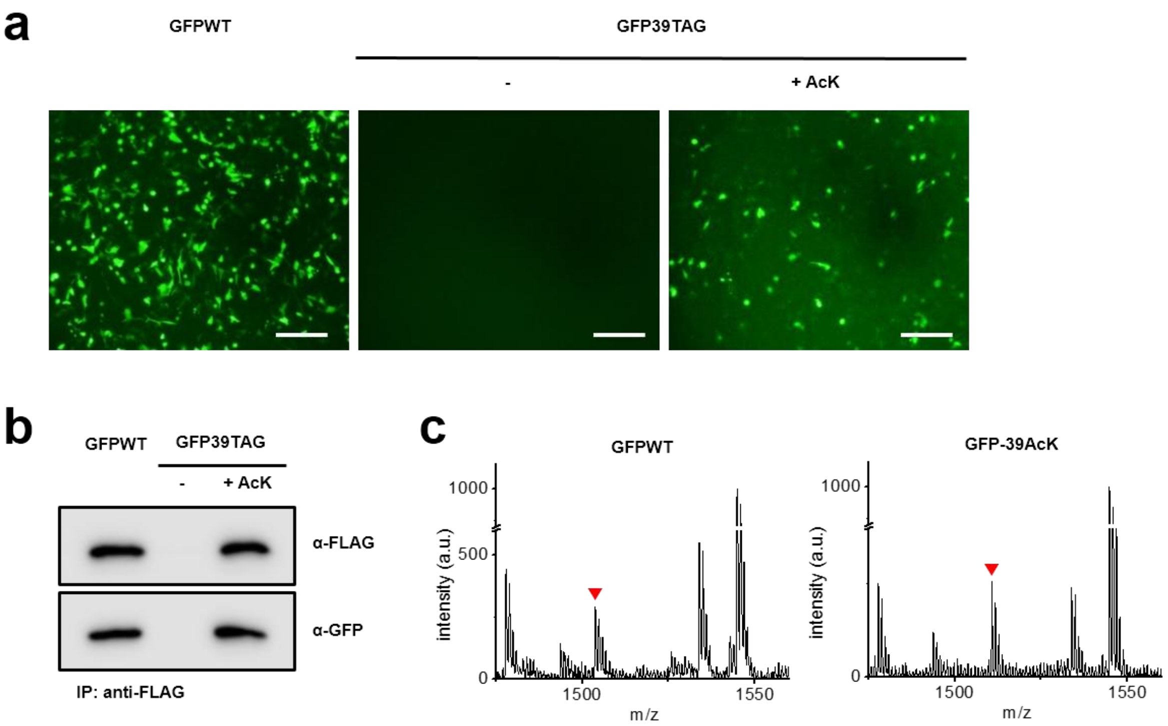

Supplementary Figure 5. Site-specific incorporation of AcK in NIH3T3 mouse cells. (**a**) Fluorescence image of NIH3T3 cells transfected with DNA constructs for AcK incorporation in the presence or absence of AcK. Scale bar, 200μm. (**b**) Western blot analysis using antibodies specific for FLAG-tag and GFPuv. For immunoblotting, proteins in the lysates of the transfected cells were pulled down with antibody against FLAG-tag and resolved by SDS-PAGE. (**c**) MALDI-TOF MS analysis of wild type GFPuv (GFPWT) and AcK-incorporated GFPuv (GFP-39AcK) after trypsin digestion. The peak corresponding to the tryptic peptide carrying Y39 of GFPWT or AcK39 of GFP-39AcK is indicated (arrowhead). The mass of Y39-containing tryptic peptide is 1,503.8 Da (calculated mass; 1,503.7 Da). The detected mass of AcK39-carrying tryptic peptide is 1,510.7 Da (expected mass; 1,510.7 Da).

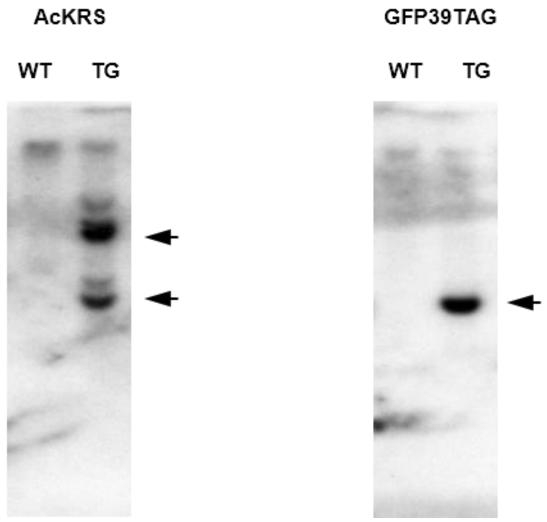

Supplementary Figure 6. Uncropped Southern blot images for Figure 2a.

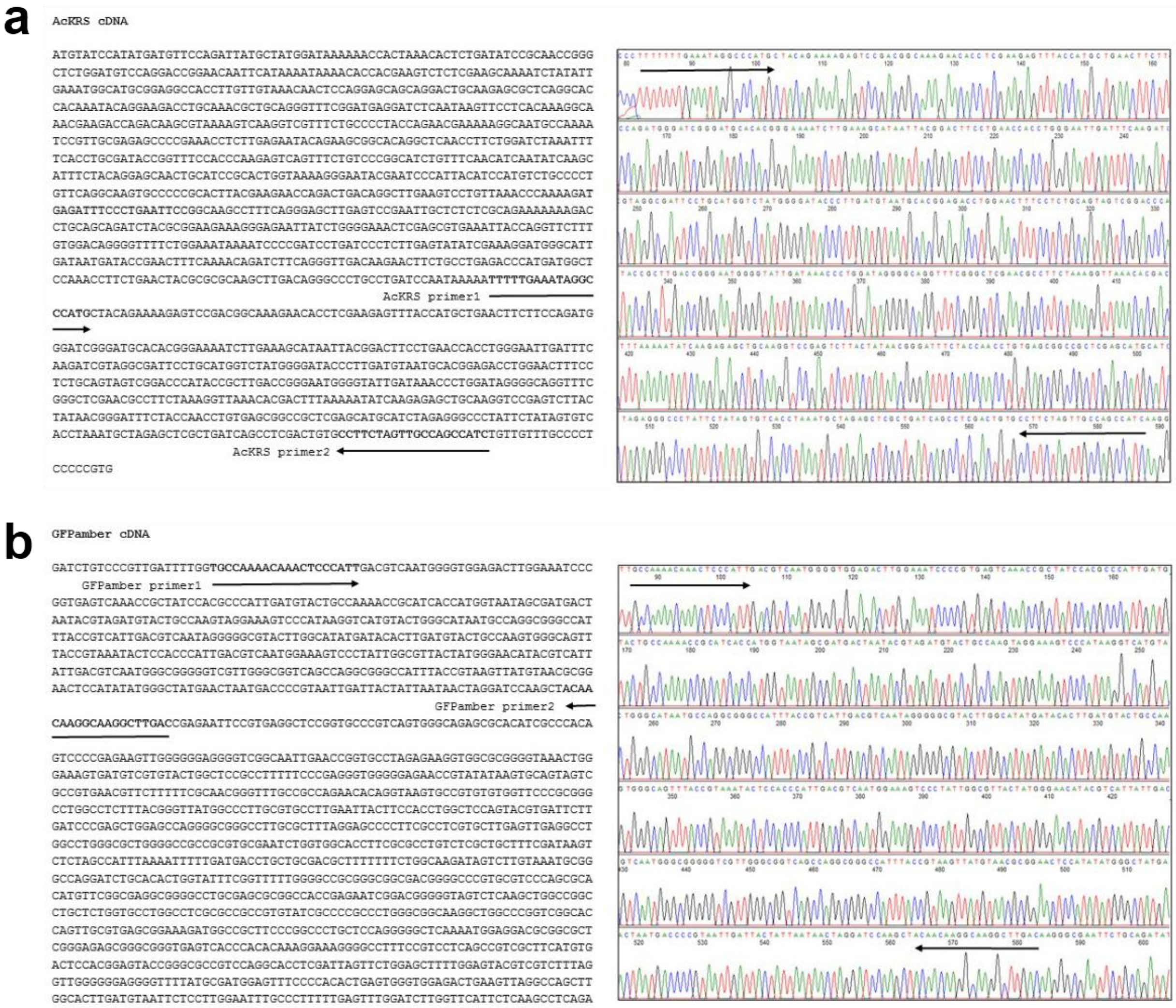

Supplementary Figure 7. Validation of genomic insertion of transgenes. Genomic DNA was isolated from the tail of AcK-GFPamber mouse and gene fragments of AcKRS and GFPamber were amplified by PCR. PCR products were cloned into TA vector (Invitrogen) and subject to sequence analysis. Sequences of AcKRS (a) and GFPamber (b) were correctly identified.

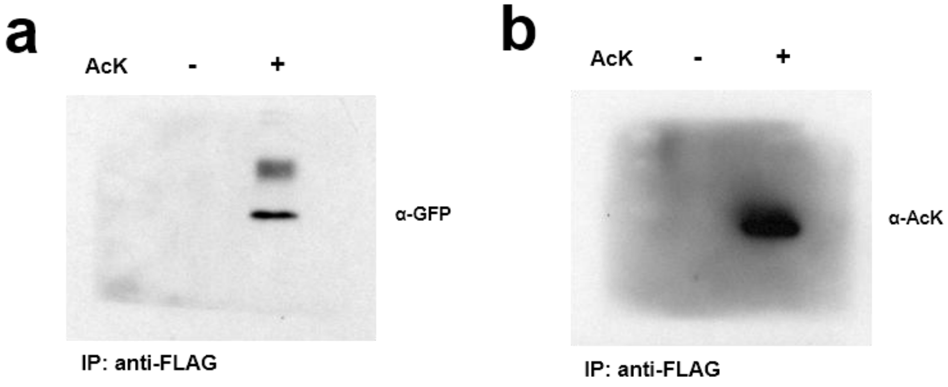

Supplementary Figure 8. Uncropped Western blot images for Figure 2d.

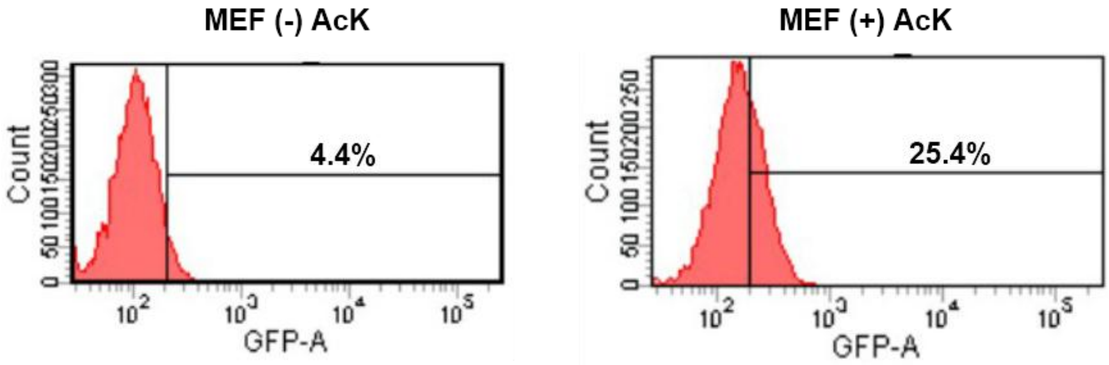

Supplementary Figure 9. Flow cytometric analysis for acetylated GFP expression in the MEF cells. The MEF cells were cultured in the presence or absence of 10 mM AcK and GFPuv expression was analyzed using flow cytometry. GFPuv-positive cells have increased from 4.4% up to 25.4% by the presence of AcK.

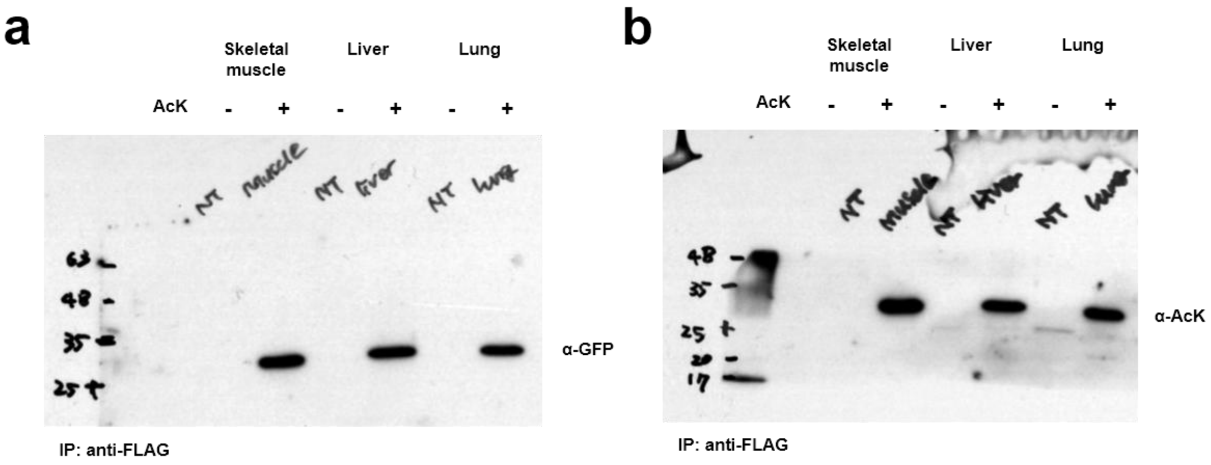

Supplementary Figure 10. Uncropped Western blot images for Figure 4b.

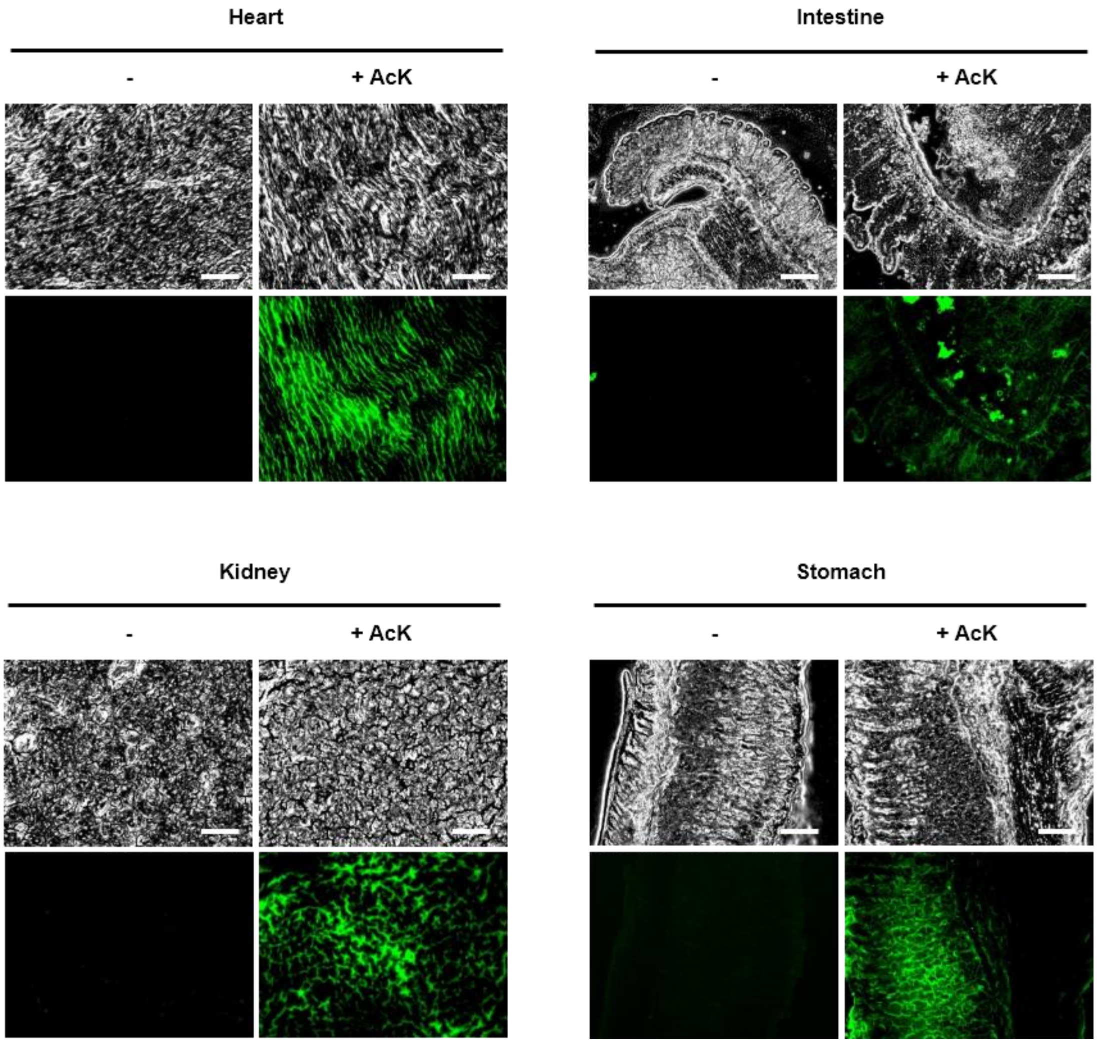

Supplementary Figure 11. Fluorescence image of tissues from AcK-GFPamber double transgenic mouse fed with AcK. Tissues from transgenic mice were frozen, cryo-sectioned, and analyzed on fluorescence microscope. The expression of GFPuv in tissues, heart, intestine, kidney, and stomach was detected only after AcK injection, demonstrating temporal expression of acetylated GFPuv in various tissues of the AcK-GFPamber mouse. Scale bar, 200μm.

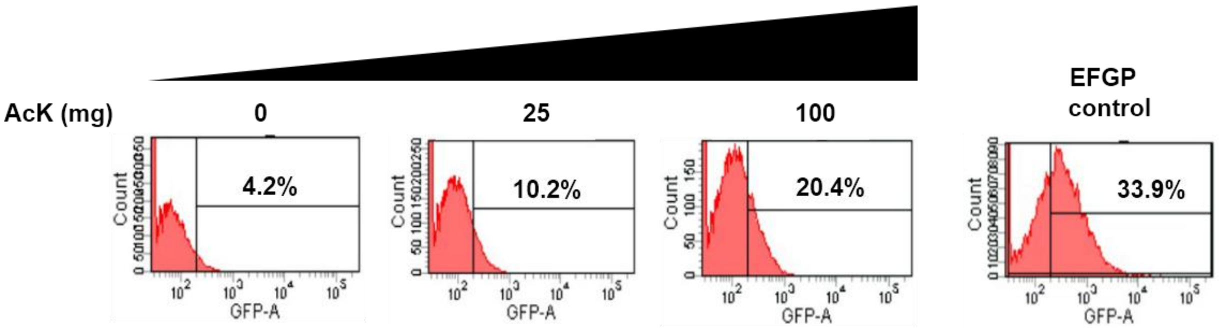

Supplementary Figure 12. Flow cytometric analysis for acetylated GFP expression in liver cells. Indicated amount of AcK was IP-injected to AcK-GFPamber. Liver cells were dissociated by dispase and collagenase and analyzed by flow cytometry. Upon addition of AcK, a dose-dependent fluorescence increase was observed. EGFP-expressing mouse strain was used as a control.

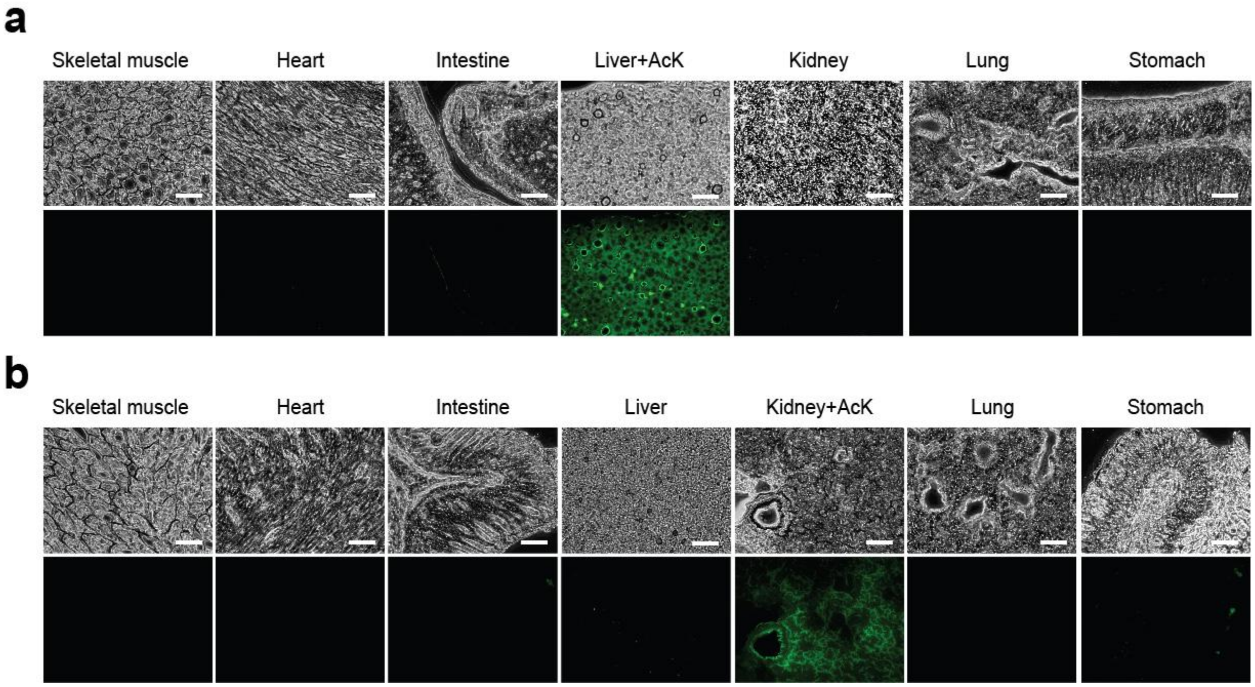

Supplementary Figure 13. Tissue specific expression of acetylated GFPuv. Tissues were collected after direct injection of AcK to target tissues and analyzed by fluorescence microscopy. Acetylated GFPuv was observed in liver (a) or kidney (b) only when AcK was directly delivered to the corresponding tissues. Scale bar, 200µm.

Supplementary Table 1. Tryptic digestion of GFPuv with a C-terminal FLAG-tag. The peptide with the modification site (Y39) is indicated in bold

Residues	Peptide sequence	Expected mass $[M+H]^+$
1-4	MESK	494.2
5-27	GEELFTGVVPILVELDGDVN GHK	2437.3
**28-42**	**FSVSGEGEGDATYGK**	**1503.7**
43-46	LTLK	474.3
47-53	FICTTGK	769.4
54-74	LPVPWPTLVTTFSYGVQCFS R	2398.2
75-80	YPDHMK	790.4
81-81	R	175.1
82-86	HDFFK	693.3
87-97	SAMPEGYVQER	1266.6
98-102	TISFK	595.3
103-108	DDGNYK	711.3
109-110	TR	276.2
111-114	AEVK	446.3
115-123	FEGDTLVNR	1050.5
124-127	IELK	502.3
128-132	GIDFK	579.3
133-141	EDGNILGHK	982.5
142-157	LEYNYNSHNVYITADK	1943.9
158-159	QK	275.2
160-163	NGIK	431.3
164-167	ANFK	479.3
168-169	IR	288.2
170-210	HNIEDGSVQLADHYQQNTPI GDGPVLLPDNHYLSTQSALS K	4472.2
211-215	DPNEK	602.3
216-216	R	175.1
217-239	DHMVLLEFVTAAGITHGMDE LYK	2590.3
240-242	DYK	425.2
243-247	DDDDK	607.2

Supplementary Table 2. Peptide fragment carrying the modification site (position 39) of wild type GFPuv or UAA-carrying GFPuv after trypsin digestion. Expected and observed mass of each tryptic peptide are shown.

**GFP**	**Tryptic peptide (170-210)**	**Mass $[M+H]^+$, m/z**	
		**Expected**	**Observed**
GFP wt	FSVSGEGEGDAT**Y**GK	1503.7	1503.7
GFP-39AcK	FSVSGEGEGDAT**AcK**GK	1510.7	1510.9
GFP-39tfAcK	FSVSGEGEGDAT**tfAcK**GK	1564.7	1564.6
GFP-39BrF	FSVSGEGEGDAT**BrF**GK	1565.6	1565.8
